# Analysis of β-globin Chromatin Micro-Environment Using a Novel 3C Variant, 4Cv

**DOI:** 10.1371/journal.pone.0013045

**Published:** 2010-09-29

**Authors:** Ryan C. Pink, Christopher H. Eskiw, Daniel P. Caley, David R. F. Carter

**Affiliations:** School of Life Sciences, Oxford Brookes University, Oxford, United Kingdom; Tulane University Health Sciences Center, United States of America

## Abstract

Higher order chromatin folding is critical to a number of developmental processes, including the regulation of gene expression. Recently developed biochemical techniques such as RNA TRAP and chromosome conformation capture (3C) have provided us with the tools to probe chromosomal structures. These techniques have been applied to the β-globin locus, revealing a complex pattern of interactions with regions along the chromosome that the gene resides on. However, biochemical and microscopy data on the nature of β-globin interactions with other chromosomes is contradictory. Therefore we developed a novel 4C variant, Complete-genome 3C by vectorette amplification (4Cv), which allows an unbiased and quantitative method to examine chromosomal structure. We have used 4Cv to study the microenvironment of the β-globin locus in mice and show that a significant proportion of the interactions of β-globin are inter-chromosomal. Furthermore, our data show that in the liver, where the gene is active, β-globin is more likely to interact with other chromosomes, compared to the brain where the gene is silent and is more likely to interact with other regions along the same chromosome. Our data suggest that transcriptional activation of the β-globin locus leads to a change in nuclear position relative to the chromosome territory.

## Introduction

The way in which mammalian genomes compress into a nucleus approximately 200,000 times smaller is unknown. Researchers agree that, at the first level of folding, DNA wraps around histone proteins to form nucleosomes. It is less clear, however, how nucleosomes are arranged into higher-order structures such as chromosomes. Understanding this at a genome-wide scale is critical as chromosomal structures are linked to various nuclear functions including the control of gene expression, replication, DNA repair and cell division [1], [2], [3], [4]. The physical arrangement of chromosomes relative to one another, and how this relates to function of genes, is a matter of debate [5]. Much of what is known about higher-order chromatin structure comes from a variety of biochemical and imaging techniques. However, the inherent properties of these techniques, and of chromatin itself, complicate the interpretation of the results obtained.

Mounting biochemical evidence supports the idea that chromosomes form loops that are attached to a subnuclear structure or other parts of the same chromosome [6], [7], [8], [9]. Recent innovative techniques have given us the ability to detect interactions between chromatin elements, providing further evidence for the existence of looped chromatin. RNA tagging and recovery of associated proteins (RNA TRAP) uses an *in situ* hybridization protocol to deposit biotin onto chromatin fragments in the vicinity of an active gene; recovery of these tagged fragments followed by real-time PCR reveals the chromatin microenvironment of the active gene [10]. RNA TRAP was used to show that the *Hbb-b1* gene (encoding β-globin) is in proximity to the locus control region (LCR) in murine nuclei, suggesting a functional interaction and potentially the base of a chromosomal loop [11]. Chromosome conformation capture (3C) and methods derived from it involve the cross-linking of chromatin with formaldehyde, DNA restriction into smaller fragments and ligation of chromatin elements cross-linked together due to their proximity in the nucleus [12], [13], [14], [15], [16], [17], [18]. The use of 3C in mammalian cells confirmed the interaction between *Hbb-b1* and the LCR, and in addition revealed the physical proximity of both with other regulatory elements in a structure termed the active chromatin hub [13].

Indeed, with the development of these tools the list of reported chromatin interactions is increasing. The onus now is to understand the functional basis of these interactions in the context of chromosomal structure and genomic regulation. A large body of evidence points to the act of transcription and transcriptional machinery in establishing and maintaining chromatin interactions [3], [19], [20]. The clustering of transcriptional machinery would ensure high local concentrations of RNA polymerase and is likely to occur spontaneously in a crowded nucleus through depletion-attraction [21]. The notion of transcription acting as the tie between loops is supported by 3C studies; the *Hbb-b1* gene interacts with the LCR in liver cells where both are transcriptionally active but not in the brain where both are silent [13]. *Uros* and *Eraf* are two genes located over 20 Mbp away from *Hbb-b1* on the same chromosome; 3C detects a physical proximity between all three genes that is dependent on transcriptional activity, suggesting they may share the same transcription factory [14], [22], [23]. Taken together these results lead to a model of chromosomal structure, in which transcription factories are required to dynamically segregate chromatin into loops [7].

Further evidence for the importance of transcription in driving chromosomal structure comes from results using derivatives of 3C, known collectively as 4C [23], [24], [25], [26], [27], [28], [29], which show a tendency for active transcription units to cluster together.

Fluorescence microscopy experiments using chromosome-paint probes reveal that chromosomes occupy discrete territories within the nucleus [30], but the precise nature of these territories is unclear [5], [31]. Transcriptional and RNA processing machinery can be observed at the surfaces of territories, leading to a model in which active genes loop away from the main body of the chromosome to be transcribed in the interchromosome domain [32]. Indeed, some genes appear to reposition away from territories upon transcriptional activation [33], [34], [35]. However, other studies suggest that not all active genes emanate from territories [36], and that transcriptional status is not related to nuclear position relative to the territory [37].

The nuclear dynamics of the β-globin gene-cluster and how this relates to transcriptional regulation also remain unresolved. In human cells in which β-globin is transcriptionally inactive the gene can be observed towards the periphery of the chromosome territory [38], [39] and associated with repressive pericentric heterochromatin [40]. In human erythroid cells expressing β-globin there is no evidence that the gene specifically moves away from the chromosome territory upon activation [36], but it no longer co-localises with pericentric heterochromatin [40]. Experiments on the murine β-globin locus suggest that during early erythroid differentiation the *Hbb-b1* is in a poised state at the periphery of the nucleus and away from the chromosome territory [41], [42]. Later in erythroid development, when the gene becomes active, it moves closer to the body chromosome and away from pericentromeric heterochromatin at the nuclear periphery [41], [42]. These results are contradicted by another study, which showed no extrusion of *Hbb-b1* from the territory, either before or after transcriptional activation [36]. Transcription does seem to play a role in β-globin spatial dynamics, as do enhancers (but these may be through the activation of transcription) [42]. 4C data on the β-globin locus has yielded contradictory evidence regarding the importance of interchromosomal interactions [23], [29].

Here we present a novel method, which we call Complete-genome 3C by vectorette amplification (4Cv), that allows identification of physical associations of a genomic region. The method relies on the cloning and sequencing of 3C-mediated ligations to a specific sequence, and therefore is not biased towards a limited set of potential chromosomal interactions. Our data show the potential of this technique to assay genomic structure, and suggest that the β-globin gene may interact with other chromosomes more frequently during transcriptional activation.

## Results

### Complete-genome 3C by vectorette amplification

**Figure 1:**
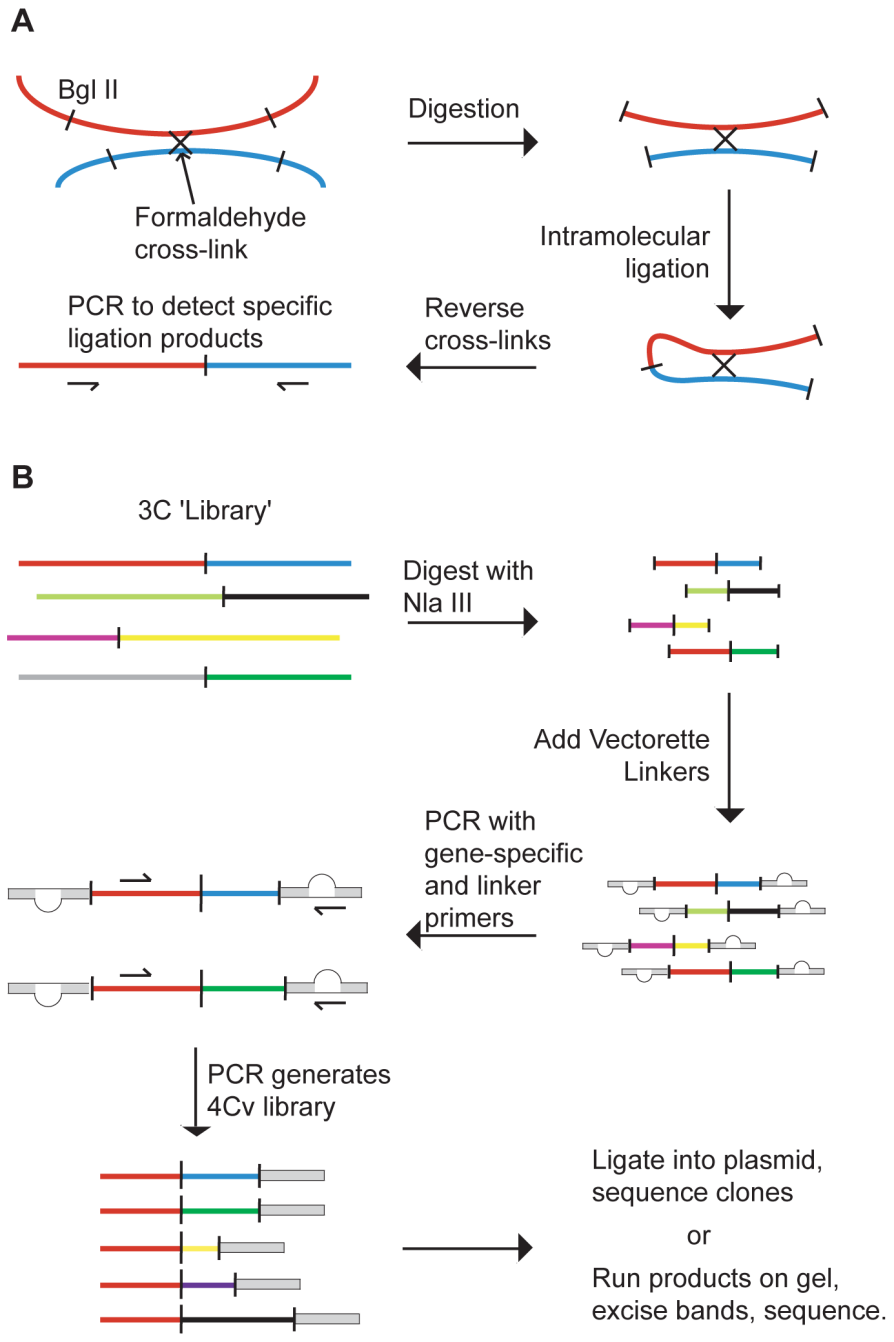
Schematic representations of Chromosome Conformation Capture (3C) and Complete-genome 3C by Vectorette amplification (4Cv). A: 3C - The physical proximity between two DNA elements in cells is converted into a direct juxtaposition, which is detected by PCR using sequence-specific primers. B: The library of genomic interactions is digested with a second enzyme (NlaIII), vectorette linkers are added and the subset of interactions involving a specific sequence (e.g. β-globin, red line) is amplified. The resulting 4Cv library is analysed by sequencing cloned products or bands excised from a gel.

3C involves the ligation of chromatin that has been formaldehyde cross-linked and restriction digested, converting a physical proximity between two genomic elements into a direct juxtaposition (Figure 1A) [12]. Specific spatial associations, captured as ligated DNA species, can be detected by PCR using pre-designed primers. Interrogation of 3C products in this manner, however, only accesses a small proportion of the information potentially available. Indeed, the 3C reaction captures a multitude of interactions representing a range of chromosomal structures in a population of cells. Conventional 3C assesses the relative cross-linking frequency between two genomic elements using PCR with pre-designed primers. To increase throughput and overcome the limitation of *a priori* knowledge of the two potentially interacting elements a range of 3C variants, collectively known as 4C, have been recently developed. They utilise PCR strategies to amplify sequences ligated to a specific genomic ‘anchor point’. We developed a novel 4C variant, Complete-genome 3C by vectorette amplification (4Cv), to assess the contribution of inter-chromosomal interactions to the transcriptionally active or silent β-globin microenvironment.

The 4Cv methodology is shown in Figure 1B. Following a 3C reaction, the DNA library is digested with NlaIII and vectorette linkers are ligated to the resulting 3′ overhangs. Vectorette linkers are approximately 54 bp long and are double stranded across most of their length, with a region of non-complementarity at the centre. PCR amplification is performed with primers specific to the linker and the genomic anchor point. The vectorette primer is complementary to the region absent in the ‘bubble’ of the linker, and therefore cannot prime until polymerase extends from the anchor point and through the linker. This prevents the vectorette primer from non-specifically amplifying all products in the 3C library. Therefore the 4Cv reaction amplifies all of the ligation products in a 3C library associated with a specific genomic anchor point.

### 4Cv amplifies a subset of the 3C library

**Figure 2:**
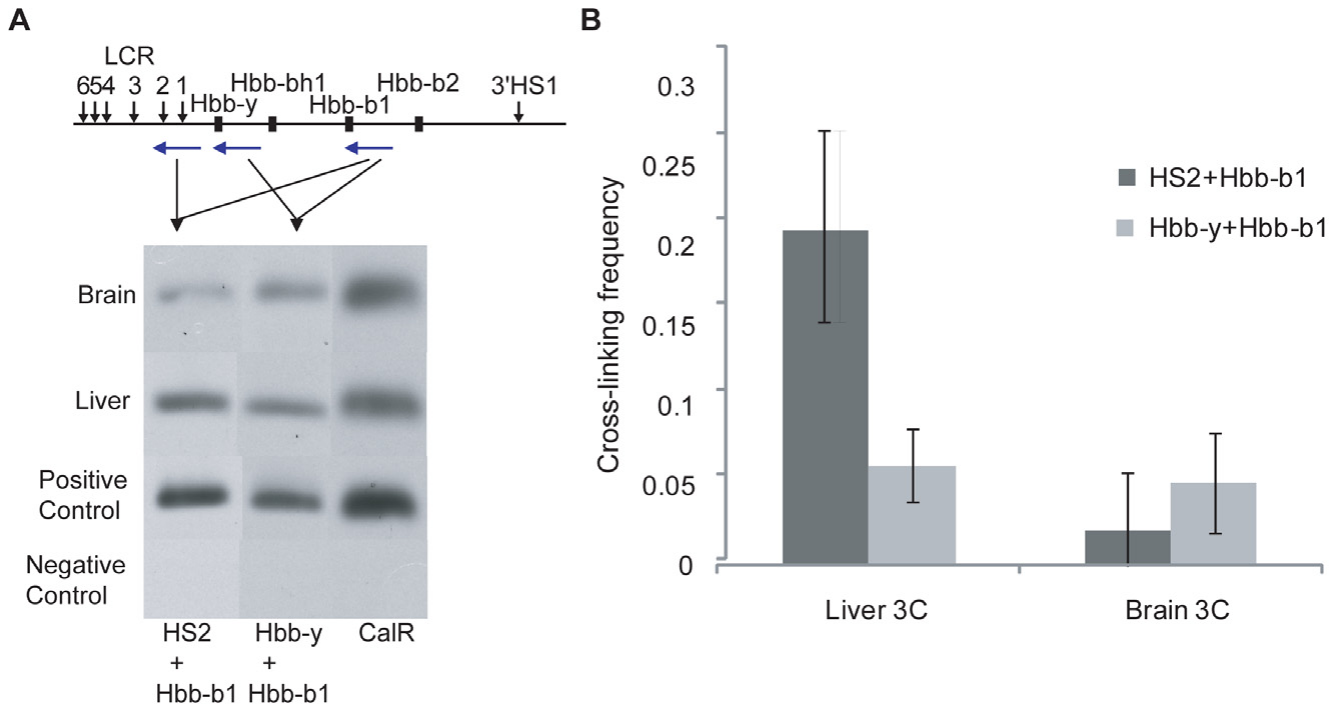
3C results confirm the specific interaction between LCR and active gene. **A**: 3C was performed in fetal liver and brain, and ligation products detected using the primer pairs shown. Primers for Calreticulin (CalR) were used as a control for cross-linking. Positive and negative controls were an equimolar mix of all PCR products and water respectively. Black squares represent genes, short arrows represent hypersensitive sites, horizontal purple arrows represent approximate primer positions (not to scale). B: Real time PCR using SYBR green was used to accurately quantify levels of *Hbb-b1* ligated to HS2 and *Hbb-y* following 3C reactions on fetal liver and brain samples. Cross-linking frequency was determined relative to CalR (see materials and methods). HS2-*Hbb-b1* cross-linking frequency is shown as a dark grey box, and *Hbb-y*-*Hbb-b1* as a light grey box. Standard deviation from at least 3 independent experiments is shown.

To provide proof of concept for the 4Cv technique we amplified sequences ligated to the murine β-globin gene, *Hbb-b1*. 3C was performed using fetal liver and brain cells from E18.5 mice; in the latter the *Hbb-b1* gene is silent and the β-globin locus is in an extended conformation, whereas in the former the active gene and LCR interact to form a higher-order chromatin structure [11], [13]. We first confirmed by quantitative RT-PCR that *Hbb-b1* is essentially silent in fetal brain and active in fetal liver (not shown). To ensure that the 3C libraries we generated represent a true reflection of nuclear interactions in E18.5 cells we used PCR to confirm the functional LCR-β-globin interaction. PCR with primers for *Hbb-b1* and HS2 (a hypersensitive site of the LCR) generates a clear ligation product in 3C from liver cells (Figure 2A). Despite the greater proximity of *Hbb-y* to the *Hbb-b1* gene on the chromosome sequence, primers specific to *Hbb-b1* and *Hbb-y* genes produced a weaker PCR product in the liver 3C library (Figure 2A). In contrast, the brain 3C library produced more *Hbb-b1*-*Hbb-y* product than *Hbb-b1*-LCR product (Figure 2A). Whilst this suggests an increase in the relative level of HS2-*Hbb-b1* interaction in the liver it does not confirm a looping structure *per se.* We therefore quantified the relative levels of ligation products compared to a Calreticulin (CalR) 3C product in E18.5 liver and brain using SYBR green real-time PCR, confirming the expected pattern of interactions (Figure 2B). These results reflect previously observed observations in the globin locus and confirm the validity of using the 3C library in a 4Cv reaction.

**Figure 3:**
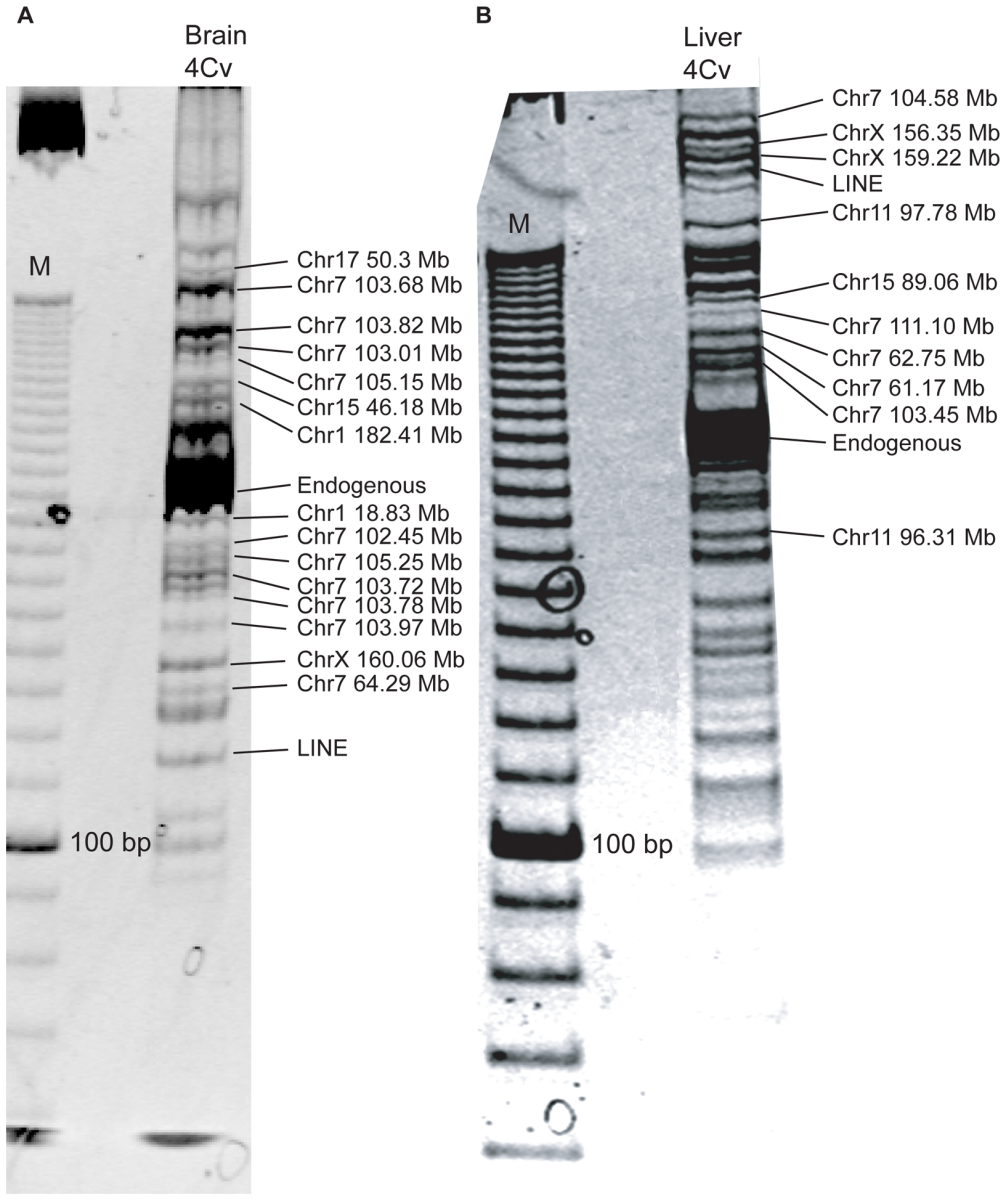
Typical result of the 4Cv procedure. 4Cv was performed by amplifying ligation products containing *Hbb-b1* from an E18.5 brain (A) or liver (B) 3C library. 4Cv products separated on a 15% polyacrylamide gel next to a 10 bp ladder (M). Bands were excised, reamplified and sequenced, chromosomal locations of the *Hbb-b1*-ligated sequences are indicated on the right. Sequencing results showed that the band at approx 205 bp was the original ‘endogenous’ sequence at the *Hbb-b1* gene (obtained when BglII fails to cut during the restriction digest stage of 3C).

4Cv was performed as described above, and ligation products containing the β-globin gene were amplified using an *Hbb-b1* primer. Gel electrophoresis revealed a mixture of products differing in size (Figure 3). To identify sequences within the mixture we employed two methods. Firstly, large quantities of 4Cv product were separated on a gel and the bands were excised, re-amplified, and sequenced directly. Figures 3A and 3B display the result of sequencing of excised bands for 4Cv experiments amplified with the *Hbb-b1* primer using brain and liver respectively. Note that the most intense product is the ‘endogenous’ genomic sequence of *Hbb-b1*, produced when the original genomic sequence is not digested by BglII during the 3C reaction. The intensity of this band masked the sequence of similarly-sized bands. Secondly, PCR product was digested, ligated into plasmid, cloned and individual products sequenced. The results from at least two independent experiments were pooled (regions that could be uniquely mapped to the mouse genome are presented in Table S1). The majority (average of 58% for brain and liver) of captured interactions were sequences on the same chromosome as *Hbb-b1* (chromosome 7). Surprisingly we observed no products representing self-circularization of the anchor fragment. Statistical analyses demonstrated that the number of chromosome 7 interactions for both liver and brain where significantly higher than expected by random (p<<0.001, Chi-square test), suggesting the interactions captured are representative of physical associations. Indeed, the distribution of intrachromosomal interactions was non-randomly distributed, with the majority in liver and brain clustered within 5 Mb of the β-globin locus (p<<0.001, Chi-square test). This suggests that the results are not caused by cross-linking artifacts and that 4Cv is able to capture chromatin sequences in proximity to the β-globin locus.

### Differences in the microenvironment of the active and inactive β-globin gene

Comparison of the results of 4Cv using liver and brain reveals some interesting differences, giving insight into the effect of transcriptional status on the microenvironment of the *Hbb-b1* gene. Analysing the proportion of endogenous sequence products in the library should give insight into the sensitivity of the *Hbb-b1* gene to restriction digestion. 4Cv libraries from 18.5E brain cells contained a higher proportion of the endogenous sequence than those from 18.5E liver cells (53% compared to 42%). This suggests that the *Hbb-b1* gene is less accessible to restriction digestion during 3C in the brain where the gene is silent, in agreement with previous work demonstrating a link between nuclease accessibility and gene activity in the globin locus [43], [44].

**Figure 4:**
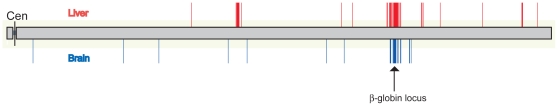
Distribution of 4Cv results on chromosome 7. Sequencing analysis of 4Cv experiments reveals the distribution of intrachromosomal interactions of *Hbb-b1* in E18.5 liver (red lines, top) and brain (blue lines, bottom) cells. The centromere and β-globin locus are marked by a black line and arrow respectively.

Plotting the pattern of intrachromosomal interactions detected with *Hbb-b1* also reveals potential differences between the active and inactive gene (Figure 4). In the liver, where the gene is active, there appears to be a bias towards interactions with regions at the telomeric end of the chromosome. In contrast, the interaction pattern of the inactive *Hbb-b1* appears to show bias towards the centromeric end. This skewed pattern of interactions in the liver and brain reflects results of a previous 4C experiment using microarrays to measure intrachromosomal interactions of the globin locus [23]. Furthermore, over 60% of putative interactions detected by 4Cv were within 500 Kb of interactions previously detected using 4C [23], [29], adding further weight to the validity of the 4Cv technique.

Interactions between *Hbb-b1* and repetitive elements could be detected in both liver and brain. The proportion of interactions with repetitive sequences was marginally higher in liver than brain (10.2% vs. 7.1%). The classification of repetitive elements was broadly similar between the two tissues, mainly comprising LINE, SINE and LTR repeats. Interestingly, the subtype of interacting elements showed some degree of tissue specificity; for example, active *Hbb-b1* was detected in proximity with several LTR ERVL elements. Thus, the subtle differences in interaction pattern may reflect the functional behaviour of the *Hbb-b1* gene in different transcriptional states.

**Figure 5:**
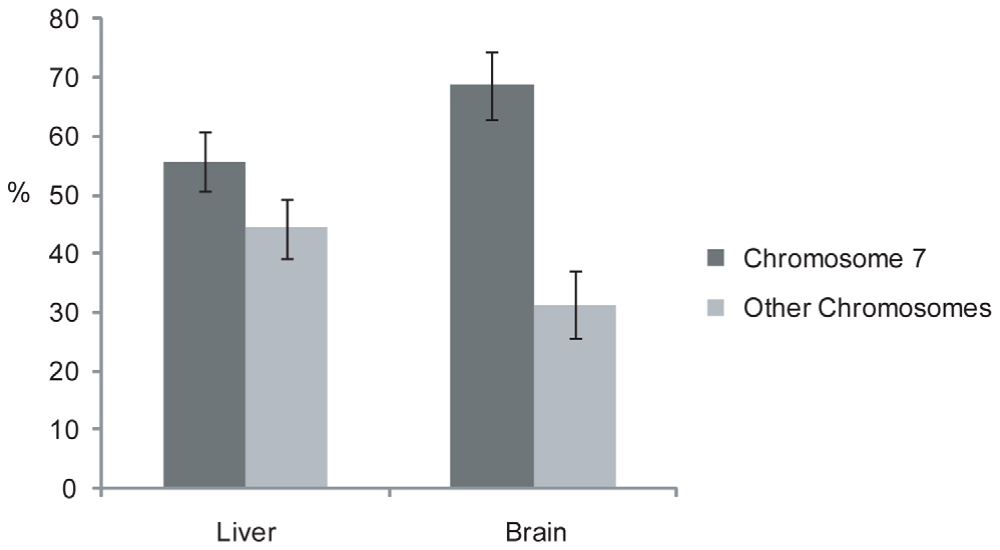
Intra- vs. interchromosomal interactions of the β-globin locus. Sequencing analysis of 4Cv experiments in fetal liver and brain reveals a greater percentage of interchromosomal (light grey) interactions when the gene is expressed (in liver). Interactions with other regions on chromosome 7 (i.e. intrachromosomal, dark grey) appear to be more prominent in the brain where *Hbb-b1* is silent. Results represent at least two biological replicates and incorporate both band and colony sequences (endogenous sequences are omitted from the calculation); error bars show standard deviation.

4Cv does not rely on prior knowledge of the potential interacting partners and is capable of detecting associations with any genomic region. The technique therefore has potential to shed light on the location of *Hbb-b1* relative to the chromosome 7 territory. The number of intra- vs. interchromosomal interactions was measured in E18.5 liver and brain cells (Figure 5). In liver cells 42% of the interactions detected for *Hbb-b1* were with regions on other chromosomes. In contrast, the inactive locus in brain cells was associated with sequences on the same chromosome in 70% of captured interactions. These differences, which are statistically significant (p = 0.034, Chi-square test), suggest that the globin locus adopts a different structure relative to the chromosome territory in active vs. inactive states. This further illustrates how differences in transcriptional activity are reflected by alterations in the nuclear environment. Collectively these results suggest that 4Cv has the potential to assay nuclear structure and detect differences in chromosomal arrangements with potentially functional significance.

## Discussion

The relationship between structure and function in the nucleus has for a long time remained something of a mystery. This is in part due to a gap in the resolution in the techniques employed to study chromatin structure. Methods such as x-ray crystallography and electron microscopy allow high resolution analysis of chromatin structure [45]. For example, recent work using electron spectroscopic imaging has revealed the molecular characteristics of transcription factories, without the use of potentially artifact-inducing heavy metals often used in staining [46]. Much of what is known about nuclear structure at coarse resolution comes from light microscopy studies. Such studies have demonstrated that the nucleus is compartmentalised, with chromosomes occupying distinct territories [30]. Consistent with this, genomic regions are able to move within discrete areas of the nucleus [47]. The higher order structures that gene loci and chromosomes adopt and the mechanisms that regulate the process are subject to intense debate.

The last decade has seen the development of several biochemical techniques that allow us to assay these structures at the required resolution [11], [12]. They have been applied to study the structure of various multi-gene clusters, including the β-globin locus, revealing a number of structural and functional interactions [11], [13], [14], [48]. Evolution of these methods has produced high-throughput variations, leading to further insight into the structure of the nucleus and how this relates to function [23], [24], [25], [26], [27], [49], [50]. Here we present the development of a novel assay, based on 3C, which we call Complete-genome 3C using vectorette amplification (4Cv). We have used 4Cv to investigate the nuclear environment of the β-globin locus in murine embryonic liver, where the β-globin genes are actively transcribed, and embryonic brain, where the gene locus is silent. 3C converts physical proximity between genomic elements into direct DNA juxtapositions. The 4Cv method amplifies a subset of the captured interactions, permitting high-throughput analysis of chromosomal regions in proximity to the β-globin locus. Our results are comparable with previous 4C methods and show that 4Cv is a valid technique for studying nuclear structure. A more thorough study will be required to assess how the different 4C techniques compare in their data output.

4Cv allows us to quantitatively compare the sensitivity to enzyme digestion at different loci, or at a genomic region under different conditions. In the case of *Hbb-b1*, our results show that the gene is more resistant to digestion in the brain, where the gene is silent. In the liver, where the gene is active, the chromatin appears to be more accessible to the 3C restriction enzyme, resulting in relatively fewer endogenous (undigested stretches of the *Hbb-b1* anchor site) sequences in the 4Cv results. Interestingly, the human β-globin locus appears to be subdivided into domains of chromatin with different susceptibility to DNase I digestion, with active genes residing in open chromatin [43]. The finding that even the active β-globin gene fails to be digested in over 40% of cases presents a financial constraint to sequencing clones as described here (approx one third of colonies sequenced yielded a useful putative interaction). This could be negated by adapting the 4Cv technique to sequence the amplified material using a high-throughput sequencing approach or further optimization of the digestion conditions. The differential digestion sensitivity of any given genomic location presents an important consideration to the interpretation of any 3C or 4C-based assay. Does the pattern of interactions seen in two tissues arise purely as a result of the availability to digestion at different sites? Whilst the results may be influenced to some degree by this factor, it is unlikely that the results entirely reflect differential sensitivity to digestion. Indeed, it is likely that observed cross-linking frequency (as assayed by 3C or 4C) of any two sequences results from a combination of their digestion availability, their spatial proximity, the frequency or duration that they are in proximity, and the proportion of cells in which they are associated. Without validating the observed 4Cv interactions using 3C and DNA FISH we cannot formally rule out the possibility that some of these represent non-functional/transient interactions that contribute to the background of all 4C experiments. However, the distribution of putative interactions detected by 4Cv is non-random and similar to those observed with previously validated 4C techniques [23], [29]. Thus, whilst we cannot make precise measurements of conformation using 4Cv it is likely that the different interactions observed for *Hbb-b1* in liver and brain, reflect their unique nuclear environments.

Our results using 4Cv to assay regions in proximity to the β-globin region in embryonic liver and brain cells are consistent with data that demonstrate tissue-specific differences in gene positioning relative to chromosome territories and other nuclear landmarks. In human lymphoid cells, where the β-globin genes are silenced, HBB is often observed at the chromosome territory periphery close to repressive pericentric heterochromatin [38], [39], [40]. Several gene loci appear to loop away from the surface of chromosome territories [31]; results in human erythroid cells, however, suggest that whilst the active β-globin locus no longer associates with pericentric heterochromatin it does not appear to move away from the chromosome body [36]. Positional analysis of the β-globin locus in mice using FISH yields different results. In one study, activation of the β-globin genes led to movement away from pericentric heterochromatin at the nuclear periphery [41], [42]. Prior to activation the locus appears to be located away from the chromosome territory, moving closer to the territory periphery upon activation [41], [42]. In contrast, a different study observed the β-globin at the territory periphery regardless of transcriptional state [36]. These contradictions could be due to the differences between mouse and human nuclear regulation, and subtleties of the experimental systems used. Where all the studies agree is that some changes in nuclear organisation do occur, and this is reflected in the 4Cv results. A previous 4C assay, based on microarray measurement of amplified 3C products, suggested that interchromosomal interactions did not contribute significantly to the nuclear microenvironment of the β-globin locus [23]. This might be expected if the β-globin locus was deeply buried in the chromosome territory; however, given the location of the β-globin locus at the territory periphery, and the finding that chromosomes often have preferred neighbours, one could speculate that a significant proportion of interactions would be interchromosomal. The lack of any significant interchromosomal interactions observed could then be due to the limited selection of chromosomes represented on the microarray or issues with sensitivity. A more recent study using enhanced 4C (e4C) showed that over 85% of the physical interactions of the active β-globin gene were with other chromosomes [29]. Our data confirm the significant extent of *trans* interactions and suggest a greater proportion of interchromosomal interactions occur in liver than in brain. This may be a result of an increased prevalence to be positioned at the surface or away from the body of chromosome territories.

Do these interchromosomal associations represent functional interactions, and if so what could drive the formation of these structures? It is conceivable that the results reflect the mingling observed for active loci at the regions between chromosomes, [15], [51], [52]. Indeed, transcription has been detected in these regions, and the recruitment of genomic regions on different chromosomes to a shared transcription factory could lead to the interchromosomal associations observed in 4Cv. This is consistent with the finding that distant active genes, on the same or even different chromosomes, can be found in proximity, possibly in transcription factories [14], [15], [53]. 4C data suggest that chromosomes dynamically fold into transcriptionally active and inactive regions [23]. The changes in nuclear localisation observed in FISH experiments could therefore be driven by changes in the occupancy of shared transcription factories. This is supported by the finding that inhibiting transcription alters the size and morphology of chromosome territory and regions of chromosome mingling [51], [54], arguing that transcription in factories is a major driving force in the arrangement of chromosomes. The apparent relocation of the β-globin away from pericentric heterochromatin is preceded by transcriptional activation, suggesting that transcription may be required for the movement [42]. Indeed, deletion of the LCR prevents this change in nuclear organisation, though it remains to be determined if this is caused by a lack of transcription due to enhancer loss or a separable activity of the LCR [41], [42]. The interaction of distal genes, detected by 3C, appeared to depend on transcriptional initiation but not elongation [22]. Results from 4C show that inhibition of transcription does not lead to a loss of the compartmentalised chromosome arrangement [55]. Taken together these data suggest transcription is required to initiate, but not maintain, such structures. Our data also agree with the results of another 4C assay, showing that transcriptional induction of the HoxB1 gene in mouse cells led to a greater frequency of interchromosomal interaction [24]. Thus, whilst our results argue for a model of increased mingling with other chromosomes associated with transcriptional activation it remains to be established if the structures of local gene clusters, chromosomes, and interchromosomal arrangements are maintained or established by transcription, and if these structures are functionally separable.

## Materials and Methods

### Ethics Statement

All experimental procedures using mice were carried out under a project license (30/1791) granted from the Home Office UK.

### 3C

3C was performed on fetal liver and brain from E18.5 C57BL/6 mice. Tissue was disrupted in DMEM media supplemented with 10% fetal calf serum by aspiration through a 200 µl pipette tip and then by passage through a 70 µM sieve. 2×10^7^ cells were resuspended in 50 ml of DMEM supplemented with 10% FCS and fixed with a final concentration of 2% formaldehyde for 7.5 minutes at room temperature; the reaction was quenched by adding glycine to 0.125 M. Cells were collected by centrifugation at 3500 rpm for 10 minutes (4°C), washed in PBS and spun again. Cells were lysed in 50 ml of lysis buffer (10 mM Tris.HCl pH 8, 10 mM NaCl, 0.2% NP-40, 0.1 mM PMSF, 1∶100 protease inhibitor cocktail [Sigma]) for 90 minutes on ice with constant stirring. Pellets of nuclear material were collected by centrifugation at 2500 rpm for 10 minutes (4°C), resuspended in 1 ml of 1.2x NEB buffer 3 with 0.15% SDS and incubated for 1 hr at 37°C with agitation. 150 µl of 20% triton X-100 was added and the mixture incubated for a further hour at 37°C with agitation. Aliquots of 1.4×10^6^ cells were resuspended in 500 µl of 1x NEB buffer 3 and digested overnight with 500 units of BglII at 37°C with agitation. BglII was inactivated by addition of SDS to 1.6% and heating to 65°C for 20 minutes. Samples were diluted in 7.5 ml of ligation buffer (30 mM Tris.HCl pH 8, 10 mM MgCl_2_, 10 mM DTT, 1 mM ATP) supplemented with 570 µl 20% triton X-100 and incubated at 37°C with gentle agitation. 6 µl of high concentration T4 DNA ligase (NEB) was added and the mixture was incubated at room temperature for 6 hours with gentle mixing. Cross links were reversed by addition of 160 µl of 10 mg/ml proteinase K and incubation at 65°C overnight. The sample was treated with RNaseA (final concentration 400 ng/ml) for 30 minutes at 37°C. DNA was isolated by phenol/chloroform extraction and ethanol precipitation and resuspended in 100 µl of molecular biology grade water.

### PCR and real time PCR

PCR reactions were performed on approximately 100 ng of 3C material. Cycling conditions were as follows: 95°C for 10 minutes, followed by 36 cycles of 95°C for 15 sec, 60°C for 30 sec and 72°C for 30 sec. Primer sequences were as follows: HS2 for – TATCACTTATTCCTCAAGTGTTGATGT; εy for – AAAGGGTTAATGCCGTGGAG; *Hbb-b1* for – CTCAGAGCAGTATCTTTTGTTTGC; CalR1 for – CTCCAGATAAACCAGTATGATCC; CalR2 for - AAACCAGATGAGGGCTGA. The CalR primers detect a 3C interaction between restriction sites approximately 1.5 Kb apart in a ubiquitously expressed region of the Calreticulin locus. The cross-linking frequency of this region is thought to be similar in fetal liver and brain [13]. PCR products were resolved on a 1.5% agarose gel and stained with ethidium bromide. An equimolar mix of all PCR products was generated and used as a positive control for PCR. Water was used as a negative control to ensure there was no PCR contamination.

Real time PCR using a SYBR green PCR mastermix (Applied Biosystems) was performed on a Corbett Rotor Gene machine with the same primer pairs described above. To quantify 3C products a standard curve for each primer pair was generated using dilutions of the equimolar PCR product-mixture. Cross linking frequencies were calculated by comparing the Ct values for a primer pair in the 3C to the control dilution series and then normalizing to the Ct values for the normalized CalR primer pair in the same 3C.

### 4Cv

Approximately 250 ng of 3C DNA was digested with NlaIII overnight at 37°C and then heat inactivated for 20 min. 4 µl (1.3 µg) of annealed vectorette linker (forward - CTCTCCCTTCTCGAATCGTAACCGTTCGTACGAGAATCGCTGGGGGATCCTTCATG, reverse – AAGGATCCCCGCGCTGTCTGTCGAAGGTAAGGAACGGACGAGAGAAGGGAGAG) was added, the sample volume was adjusted using 10x ligase buffer and 1 µl of high concentration T4 DNA ligase (NEB) was added. Ligation was performed at 16°C for 4 hours, followed by heat inactivation at 65°C for 20 minutes. Nested PCR was performed to amplify 3C products containing the *Hbb-b1* anchor region. First round PCR was performed with the 3C *Hbb-b1* For and Vectorette Primer 2 (TAACCGTTCGTACGAGAATCG) primers using Pfu Taq and the following cycling parameters: 95°C for 5 min, followed by 18 cycles of 95°C for 15 sec, 58°C for 30 sec and 70°C for 2 min, followed by a final step of 70°C for 4 min. Samples were cleaned using a Qiagen PCR purification kit and resuspended in 30 µl of molecular biology grade water. A scaled-up (96 wells with 10 µl of 1∶1000 PCR product from the first round per reaction) PCR with the nested primers *Hbb-b1* Anchor BamHI (TAGGATCCAGGTAGAACCCTTGCCTGTTTT) and Nested Vectorette Primer (ACCGTTCGTACGAGAATCGCT) was performed using the same conditions as the first round PCR except 30 cycles of PCR were used. PCR products were pooled and cleaned using a PCR purification kit (Qiagen).

### Cloning and sequencing of 4Cv products

PCR products were digested with BamHI and ligated into BamHI-digested pZero plasmid (Invitrogen) and transformed into electrocompetent E. coli cells (Invitrogen) by electroporation following the manufacturer's instructions. Colonies with inserts were picked and plasmid DNA was amplified directly using the PlasmidAmp kit (Qiagen). Insert presence was verified by agarose gel electrophoresis and DNA was sent to Geneservice for sequencing.

PCR products were also identified by excising from a polyacrylamide gel, re-amplifying and sequencing. 4Cv products were resolved using a large 7% polyacrylamide gel, stained with ethidium bromide and visualized on a UV transilluminator. Bands were cut from the gel and transferred to a 0.5 ml tube with a hole at the bottom (created using a fine bore needle). The 0.5 ml tubes were placed in 1.5 ml tubes and centrifuged for 2 minutes at 10,000 g, shredding the gel slice into a fine powder. 150 µl of LoTE (3 mM Tris.HCl pH 7.5, 0.2 mM EDTA)/7.5 M ammonium acetate (5∶1 ratio) was added and the mixture was briefly vortexed and incubated at 65°C for 2 hours. The mixture was transferred to a filter column (0.45 µM durapore filter, Millipore) and centrifuged for 3 minutes at 10,000 g. DNA was ethanol precipitated using glycogen as a carrier and resuspended in 20 µl of molecular biology grade water. The DNA was diluted 1∶300 in water and reamplified by PCR using the primers ‘Globin nested 2’ (GTAGAACCCTTGCCTGTTTTTT) and ‘Nested Vectorette Primer’. Products were cleaned using a PCR purification kit (Qiagen) and sequenced using ‘Short Vectorette Primer’ and/or ‘Globin nested 2’ as the sequencing primer.

## Supporting Information

Table S1 Regions associated with Hbb-b1 as assayed by 4Cv in liver and brain tissue.
(0.06 MB XLS)
